# Supplementary material for: Galvanic vestibular stimulation alters the sense of upright
Source: Exp Brain Res. 2025 Nov 14;243(12):246. doi: 10.1007/s00221-025-07193-7 (PMC12618296; doi:10.1007/s00221-025-07193-7)
Supplement: Supplementary file 1 — Supplementary Material 1 [file 221_2025_7193_MOESM1_ESM.docx]

**Supplementary Material**

**Galvanic vestibular stimulation alters the sense of upright**

Sofia Müller-Wöhrstein, Hans-Otto Karnath

Center of Neurology, Division of Neuropsychology, Hertie Institute for Clinical Brain Research, University of Tübingen, Tübingen, Germany


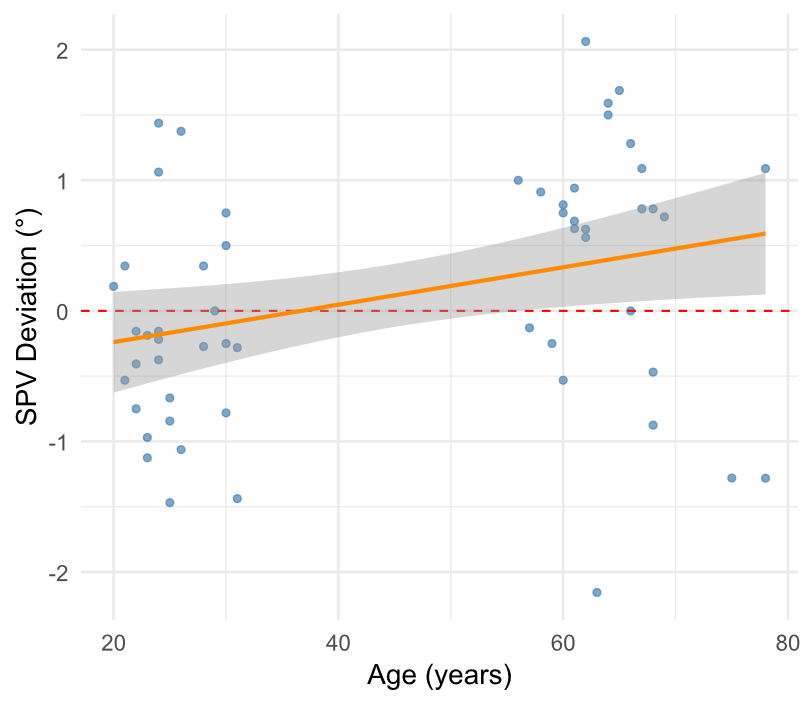


**Fig. S1** **Deviation of the Subjective Postural Vertical (SPV) as a function of age under the sham condition**

The scatter plot shows the deviation of the SPV from objective 0° as a function of the age of the entire sample under the sham condition of Galvanic Vestibular Stimulation (GVS). Blue dots show individual data points, while the orange line represents the regression line (*r* = 0.31, *p* = 0.021) with its confidence interval.
